# Association between Depression Severity and Physical Function among Chinese Nursing Home Residents: The Mediating Role of Different Types of Leisure Activities

**DOI:** 10.3390/ijerph19063543

**Published:** 2022-03-16

**Authors:** Meng Zhao, Yaqi Wang, Shan Wang, Yuan Yang, Ming Li, Kefang Wang

**Affiliations:** School of Nursing and Rehabilitation, Cheeloo College of Medicine, Shandong University, Jinan 250012, China; zhaomeng@email.sdu.edu.cn (M.Z.); wangyq@sdu.edu.cn (Y.W.); wangshan2912@163.com (S.W.); yang26happy@163.com (Y.Y.)

**Keywords:** physical function, depression, leisure activities, nursing homes, older adults

## Abstract

Despite strong evidence associating depression with poor physical function, the underlying mechanisms of this association remain unknown. This study aimed to ascertain whether different types of leisure activities mediate the effect of depression severity on physical function. This cross-sectional descriptive study included 353 Chinese nursing home residents (aged ≥ 60 years; 197 female) from nursing homes of five districts (Lixia, Tianqiao, Huaiyin, Shizhong, and Licheng) in Jinan, Shandong Province, China, from March to June 2018. Multiple-mediation analyses, including regression and bootstrap analyses, were conducted to evaluate the association of depressive symptoms’ severity and active or passive leisure activities with physical function. Active, but not passive, leisure activities significantly mediated the effect of moderate/severe depressive symptoms on physical function compared to those without depressive symptoms. In contrast, there was no significant association between mild depressive symptoms and physical function. This study demonstrates that leisure activities affect the association between depressive symptoms’ severity and physical function, and its protective role depends on the type of leisure activities. Interventions for physical function should be designed by focusing on active leisure activities among older adults, especially those with moderate/severe depressive symptoms, to delay physical function and improve overall well-being.

## 1. Introduction

The older population is rapidly increasing globally. For example, in China, the proportion of those aged ≥ 60 years has increased by 5.44% from 2010 to 2020, according to the seventh national census in 2021 [1]. Maintaining an optimal level of physical function is essential for healthy aging. Physical function is defined as integrating and converting physiological stimuli into a muscular action [2], such as walking, sitting, standing, and balancing. A decline in physical function can lead to increased risks of disability, cognitive impairment, frailty, and a decreased quality of life and a significantly increased possibility of mortality [3,4,5]. Compared with other populations, the health status of older adults residing in nursing homes are worse due to the high prevalence of physical function decline, multimorbidity, and polymedication [6]. Providing the optimal treatment for this population represents a vast challenge, particularly with the anticipated increase in the number of people residing in nursing homes. Therefore, identifying potentially modifiable risk factors associated with the loss of physical function to develop effective prevention strategies among nursing home residents is a significant public health priority, considering the aging population.

Mental health disorders, particularly depression, are a leading cause of functional decline globally [7,8,9]. A six-year, population-based, longitudinal study of community-dwelling older adults showed that depression was significantly associated with impaired physical function and chance of recovery from impaired physical function even after adjusting for demographic and various health factors [7]. Another longitudinal study conducted over four years discovered that men experiencing depression were four-times more likely to experience a rapid functional decline [8]. Thus, ample evidence suggests that depression is strongly associated with physical function [7,8,9,10,11]; however, the underlying mechanisms have not been elucidated.

One possible mechanism underlying the link between depression and physical function is leisure activity participation. Leisure activities have attracted significant attention given their low cost, accessibility, and beneficial effect on older adults’ physical and mental health [12]; hence, engaging in leisure activities may be related to physical function. Nevertheless, those findings are inconsistent. For instance, several studies reported that individuals with moderate to high physical activity levels have markedly better physical function than their sedentary counterparts [13,14,15]. However, other light leisure activities (e.g., lying in the sun, listening to the radio, and reading newspapers/books) had nonsignificant effects on physical function [16,17]. Therefore, not all leisure activities can prevent or delay physical function decline; moreover, the association may depend on the type and level of activity. Hence, identifying the types of leisure activities and exploring their effects on physical function may provide essential insights for implementing individualized interventions. Based on the results of previous studies [13,14,15,16,17], most activities with negligible effects on physical function are sedentary (sitting or lying down) in nature and involve limited energy expenditure. Conversely, activities involving physical movement delay the decline in physical function. Therefore, leisure activities are categorized into active and passive types. Active leisure activities include physical movement and exercise, either within a group or alone, while passive leisure activities are usually sedentary and involve limited to no physical movement [18,19]. The classification of leisure activities is generally intended to identify substitutable activities with common elements, such that engaging in any activity within a particular category would produce a similar effect [20].

In addition, several studies have shown that feelings of discouragement and hopelessness may reduce the willingness to participate in leisure activities [21,22,23,24]; thus, it is suggested that leisure activities may also be related to depression.

## 2. Research Hypotheses

As indicated above, depression has a significantly negative impact on physical function, and it also has a significantly negative effect on the participation in leisure activities. However, few studies have described the association between different leisure activity patterns (i.e., active and passive leisure activities) and physical function. Additionally, it is also not clear whether active and/or passive leisure activities serve to mediate the relationship between depression and physical function. Hence, this study aimed to explore the relationship between depression, different leisure activity patterns (i.e., active and passive leisure activities), and physical function among Chinese nursing home residents. We hypothesized that (1) active, rather than passive, leisure activities may be associated with physical function and that (2) active, rather than passive, leisure activities play mediating roles in the relationship between depression and physical function.

## 3. Materials and Methods

### 3.1. Participants

This cross-sectional, descriptive study was conducted among the residents of nursing homes in five districts (Lixia, Tianqiao, Huaiyin, Shizhong, and Licheng) in Jinan, Shandong Province, China, from March to June 2018. Data were collected through face-to-face interviews with older adults by trained research assistants. We initially selected 69 nursing homes from the Jinan Civil Affairs Bureau with a bed capacity of more than 30, which had operated for more than a year. A total of 42 nursing homes were excluded for the following reasons: refusal to participate (*n* = 28), relocation or renovation (*n* = 6), and missing contact details (*n* = 8). Finally, we included 27 nursing homes in this study.

**Figure 1:**
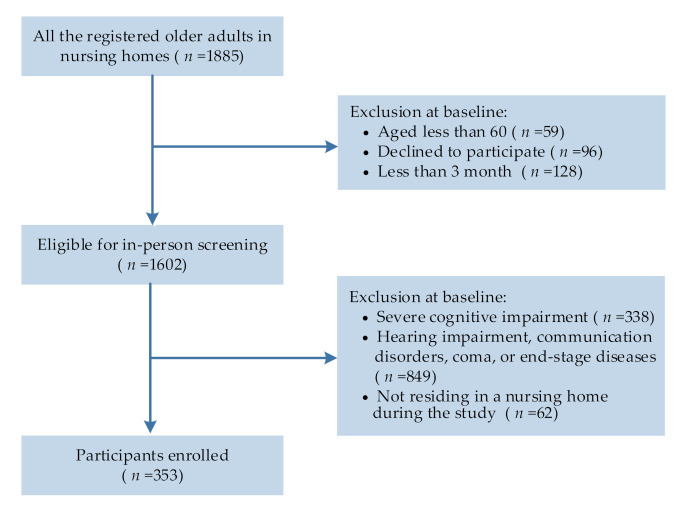
Flowchart depicting the participant selection process.

The inclusion criteria for the residents were those aged ≥ 60 years who resided in a nursing home for ≥ 3 months. The exclusion criteria were (i) hearing impairment, communication disorders, coma, or end-stage diseases; (ii) not residing in a nursing home during the study, and (iii) the presence of severe cognitive dysfunction, as determined by a Mini-Mental State Examination score <10 [25]. In total, 373 eligible residents were invited to participate. Details of the participant enrollment process are shown in Figure 1.

All participants were fully informed about the study. Completion the survey implied consent to participate in this study, which was declared to subjects at the commencement of the survey. The Ethics Committee of Shandong University approved the study (Approval number 2017-R-112).

### 3.2. Measures

#### 3.2.1. Exposures: Depression

Depression was assessed using the Patient Health Questionnaire-9 [26]. Participants were asked about the frequency of experiencing depression over the past 2 weeks. Response categories were “not at all” (scored as 0), “several days” (scored as 1), “more than half the days” (scored as 2), and “nearly every day” (scored as 3). A total score of 0–4 indicated no depression, 5–9 mild depression, 10–14 moderate depression, 15–19 moderately severe depression, and 20–27 severe depression [26]. Based on their responses to each item, the participants were categorized into three groups: no depression, mild depression, and moderate/severe depression.

#### 3.2.2. Mediators: Active and Passive Leisure Activities

An activity engagement questionnaire was used to evaluate the type of leisure activity (active or passive) [27]. Participants were asked to rate their frequency of participation in nine types of leisure activities during the prior 3 months, from 1 (never) to 5 (almost every day) [27]. Based on previous studies [18,19], we dichotomized these nine activities into active leisure activities (including singing or playing an instrument, performing physical activity, and participating in an outdoor activity) and passive leisure activities (including watching television/listening to the radio/watching theatrical performances, reading newspapers/books/magazines, writing/painting, playing cards/chess/mahjong, chatting with others, and participating in religious activities). Scores for active leisure activities (range: 3–15) and passive leisure activities (range: 6–30) were obtained by adding the individual scores for the frequency of each activity within their respective categories.

#### 3.2.3. Outcome: Physical Function

Physical function was evaluated using the Short Physical Performance Battery (SPPB) scale, comprising a standing balance test, 4-meter walking test, and a repeated chair stand test [28]. Each test was scored between 0 and 4. Subsequently, a total score ranging from 0 to 12 was obtained after summing the scores acquired in each test, with higher scores indicating better physical function.

### 3.3. Covariates

A priori, we selected potential confounders for adjustment in multivariable models based on the factors that might causally affect exposure and the study outcome. These included age (years), sex (female or male), schooling (years), having a spouse (yes or no), self-reported economic conditions (good or poor), comorbidity conditions (yes or no), and body mass index (underweight, normal, overweight, or obese). In addition, participants who reported that they were diagnosed with two or more chronic diseases were defined as having a comorbidity [29].

### 3.4. Data Analyses

Descriptive analyses (means, standard deviations, frequencies, or percentages) were performed for the whole sample and depression severity (no, mild, or moderate/severe depression). Comparisons across the depression severity categories were performed by analyzing variance for continuous variables or by performing a chi-squared or Fisher’s exact test for categorical variables.

Given that residents (Level 1) were clustered within nursing homes (Level 2), we needed to consider the necessity of using multi-level mediation. If the design effect at Level 2 was >2 and the variance between groups was significant, multi-level mediation would be required. However, the design effect was <2, and the variance between groups at Level 2 was nonsignificant (*p* > 0.05). Thus, a traditional multiple-mediation analysis was conducted according to the recommendation by Baron and Kenny [30].

We further performed a multiple-mediation analysis using the bias-corrected 95% confidence intervals (CIs), and the mediated indirect effects were calculated using 5000 bootstrap resampling [31]. A mediated indirect effect was considered significant when the CI did not include zero. The effect size was calculated to demonstrate the total effect explained by the mediators. The goodness-of-fit had a comparative fit index (CFI) > 0.95, Tucker–Lewis Index > 0.95, standardized root mean square residual (SRMR) < 0.08, and root mean square error of approximation (RMSEA) < 0.08 [32]. Analyses were performed using Mplus 7 (Muthén & Muthén, Los Angeles, CA, USA).

We performed several sensitivity analyses to evaluate the robustness of the results. These included (1) repetition of the mediation analyses adjusting only for age and sex in the models, (2) watching TV being considered as a more passive leisure activity than the other activities [33] (except for watching TV, the other six passive leisure activities require cognitive involvement and might have potential benefits for physical function [23,33]; therefore, we removed “watching television/listening to the radio/watching theatrical performances” from the category of passive leisure activities and reclassified them as cognitive leisure activities), (3) using depression as a continuous variable, and (4) using the SPPB score as the dichotomous outcome: poor physical function (<9 = 1) and good physical function (≥9 = 0) (2).

## 4. Results

Table 1 shows the participants’ characteristics. Of the 353 residents enrolled in this study, 197 were women (55.8%), with an average age of 79 years (range: 60–103). They had an average of 5 years of education. Most older adults did not have a spouse (82.2%). Approximately 60% reported having no depression, 20% reported mild depression, and 20% reported moderate/severe depression. The scores of active or passive leisure activities and physical function decreased as the reported severity of depression increased.

**Figure 2:**
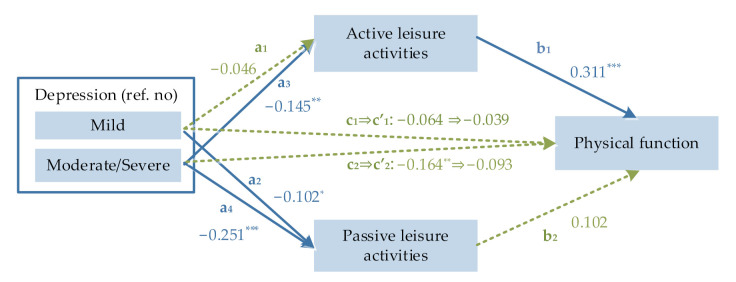
The mediating role of active and passive leisure activities in the association between depressive symptoms and physical function (*n* = 353). Standardized coefficients are shown along the path arrows; solid lines represent the statistical significance at *p* < 0.05, and dashed lines represent non-significant effects; they are adjusted for age, sex, years of schooling, having a spouse, self-reported economic conditions, comorbidity conditions, and body mass index; Path a_1_ represents the effect of mild depression on active leisure activities; Path a_2_ represents the effect of mild depression on passive leisure activities; Path a_3_ represents the effect of moderate/severe depression on active leisure activities; Path a_4_ represents the effect of moderate/severe depression on passive leisure activities; Path b_1_ represents the effect of active leisure activities on physical function; Path b_2_ represents the effect of passive leisure activities on physical function; Path *c*_1_ indicates the total effect of mild depression on physical function; Path *c’*_1_ represents the direct effect of mild depression on physical function; Path *c*_2_ indicates the total effect of moderate/severe depression on physical function; Path *c’*_1_ represents the direct effect of moderate/severe depression on physical function; * *p* < 0.05, ** *p* < 0.01, *** *p* < 0.001.

Residents who reported having moderate/severe depression had significantly worse physical function (total effect, β = −0.164; 95% CI: −0.265, −0.071) than those without depression. In contrast, there was no significant association between mild depression and physical function (total effect, β = −0.064; 95% CI: −0.063, 0.015). Moreover, residents who reported having moderate/severe depression were less likely to participate in active (β = −0.145; 95% CI: −0.256, −0.030) and passive leisure activities (β = −0.251; 95% CI: −0.347, −0.145). However, after adjusting for covariates and leisure activities, the direct effect of moderate/severe depression on physical function was rendered nonsignificant (β = −0.093; 95% CI: −0.190, 0.006). Those who were more likely to participate in active leisure activities (β = 0.311, 95% CI: 0.205, 0.412) but not in passive leisure activities (β = 0.102, 95% CI: −0.024, 0.215) had better physical function (Figure 2).

Overall, in the bootstrap analysis (Table 2), active leisure activities mediated the association between moderate/severe depression and physical function (indirect effect = −0.045, bias-corrected 95% CI: −0.083, −0.013), whereas the indirect effect of passive leisure activities was not significant (indirect effect = −0.025, bias-corrected 95% CI: −0.062, 0.003). Furthermore, active leisure activities accounted for 27% of the total effect of moderate/severe depression on physical function. However, neither active nor passive leisure activities mediated the association between mild depression and physical function. The model revealed a good fit based on CFI/TFI = 1, SRMR < 0.001, and RMSEA < 0.001.

In the sensitivity analysis, similar results were observed when using a minimally adjusted model, the category of cognitive leisure activities, and the alternative variable approach for depression and SPPB scores (see Supplementary File of Tables S1–S4 and Figures S1–S4).

## 5. Discussion

The study evaluated the underlying mediating effect of different leisure activity patterns (i.e., active and passive leisure activities) in the association between depression severity and physical function among Chinese nursing home residents. As described in the introduction, few studies have explored the underlying mechanism of this relationship between depression severity and physical function. To remedy this gap, this study was conducted to address the above research questions and found that active, rather than passive, leisure activities were associated with physical function. Our study demonstrated that active, rather than passive, leisure activities significantly mediated this association only in the most severely depressed residents (moderate/severe depression). For instance, moderate/severe depression could reduce the likelihood of engaging in active leisure activities, leading to physical function impairment. In contrast, there was no significant association between mild depression and physical function. Our findings highlighted that active leisure activities might serve as a critical target for addressing the impact of moderate/severe depression on physical function, rather than simply providing older adults with more opportunities to participate in any leisure activities.

We discovered that participants with moderate/severe depression might have a reduced probability of engaging in leisure activities than those without depression. This finding is similar to those of other studies suggesting that depression has more detrimental effects on residents’ leisure activity engagement [21,22,23,24]. Depression is one of the most prevalent mental disorders and health threats among older people worldwide. Common symptoms of depression, such as insomnia, apathy, and decreased motivation and energy, may reduce residents’ ability to participant activities [34]. Another possible reason may be that individuals with depression are more socially isolated than those without depressive symptoms [35], thus leading to a withdrawal from leisure activities. Therefore, depression negatively affects their ability to engage in leisure activities.

Our findings add to existing knowledge that active, rather than passive, leisure activities contribute to reduced functional capabilities. Previous studies also indicated that higher engagement in active leisure activities, not passive, was positively associated with higher levels of life and psychological well-being satisfaction [18,35]. The mechanisms that link active leisure activities with physical function are likely to include various behavioral, psychological, and physiological processes. Active leisure activities involve more physical movement than passive leisure activities, including local body movements, such as upper limb, lower limb, or whole-body limb movements. Such movements can reduce age-related oxidative damage and chronic inflammation and improve insulin-like growth factor−1 levels. Furthermore, they can promote anabolism, leading to an increase in muscle protein synthesis [20,36], which can affect multiple key systems, including the endocrine, immune, and skeletal muscle systems, all of which are crucial to delaying the development of chronic disease and physical limitation. Another possible explanation for our finding is that active leisure activities can enhance social relationships, self-efficacy, and positive emotions [18,35], which positively affect physical function. Previous studies found that high-intensity cognitive stimulation leisure activities have a positive effect on disability [23]. Interestingly, the sensitivity analysis found that cognitive leisure activities did not show a protective association. Indeed, nursing home residents are a relatively heterogeneous cognitive exposure group. It would be noteworthy to speculate that the cognitive stimulation from these activities was not enough to improve physical function. Ultimately, this uncertainty highlights the need for further research regarding the effect of other high-intensity cognitive stimulation activities on physical function.

When considering depression severity, we only found mediation effects of moderate/severe depression on physical function, according to Table 2. In particular, we found no mediation effects for mild depression. Our results supported this pattern, as there were no statistical differences in physical function and active or passive leisure activities between residents with mild depression and those without depression (*p* > 0.05, data are not shown).

Moreover, our results emphasized that each type of leisure activity may have a different effect on the association between moderate/severe depression and physical function. Specifically, only active leisure activities significantly mediated the association between moderate/severe depression and physical function, and no such association was found for passive leisure activities. Our findings may have important implications for the development of evidence-based prevention strategies and guidance for future practice. Notably, this study revealed that older adults preferred to participate in passive, rather than active, leisure activities. For residents with moderate/severe depression, the frequency of active leisure engagement could be as low as less than once per month. Especially during the coronavirus disease 2019 pandemic, older adults tended to have more sedentary lifestyles and less participation in active leisure activities [37]. Those factors further promoted physical limitations and increased adverse outcomes. Hence, to further reduce depression and strengthen physical function, it is necessary to initially raise awareness about the importance of active leisure engagement. Policymakers and healthcare providers should be aware of the far-reaching consequences that depression may impose on the health care system; hence, proactive planning and implementation of preventive interventions and strategies are warranted. Additionally, even for those with poor physical function, nursing homes should promote their physical involvement by organizing activities that focus on strengthening the upper extremities, such as chair yoga or other chair-based activities [38], rather than simply engaging in passive leisure activities, such as watching television. At the facility level, the management team of nursing homes needs to consider active leisure activities when preparing the schedules and caring procedures. Caregivers may periodically assess the frequency and type of leisure activities that older adults participate in, as well as the conditions that may restrict their participation in these activities. Meanwhile, the effects of these strategies could be further amplified by the nature of nursing homes. Compared with those residing in the community, older adults in nursing homes live in a collaborative environment. They share similar activities in scale and venues, which are easy to promote and benefit from, as it is easier for these measures to take effect. Older adults should engage in more active leisure activities according to their needs, interests, and abilities at an individual level.

### Limitations

Some limitations of this study have to be acknowledged. First, most of the data were self-reported and thus may be subject to recall and reporting biases. Further studies are required to corroborate the findings using objective measures, such as measuring leisure activities using accelerometers, with significant advantages in assessing the activity intensity, duration, and frequency [39]. We can further examine the mediating role of the leisure activity type (i.e., intensity, frequency, and duration) and its dose–response characteristics in the association between depression and physical function. Second, although active leisure activities explained a part of the total effect, the moderate effect size suggested that other mechanisms played a significant role in making older adults with depression more vulnerable to poor physical function. Third, our study was cross-sectional; thus, the causality between variables could not be determined. It is still possible that the results are bi-directional, because the poor physical function of residents may more likely lead them to depression, which might be associated with a reduced likelihood of engaging in leisure activities. Longitudinal studies to definitively elucidate time-sequential associations are required. Fourth, this study was conducted at nursing homes in one city in China; thus, the main findings should be confirmed in other populations or settings.

## 6. Conclusions

This study is the first to elucidate the mediating effect of depression severity on physical function among nursing home residents. Active leisure activities are critical for preventing or delaying poor physical function. Physical limitation is associated with falls, disability, mortality, and an increased need for care and health services. Therefore, it is suggested that future behavioral change-based intervention studies should be conducted to increase active leisure activities among older adults, especially among nursing home residents with moderate/severe depression.

## Figures and Tables

**Table 1:** Characteristics of the participants among nursing home residents.

Characteristic	All (*n* = 353)	Depression Severity			*p* for Difference
		No	Mild	Moderate/Severe	
		(*n* = 212)	(*n* = 70)	(*n* = 71)	
	Mean (Standard Deviations) or *n* (%)				
Age	79.01 (8.80)	79.22 (9.24)	79.60 (7.61)	77.80 (8.56)	0.415
Sex					0.184
Female	197 (55.8)	110 (51.9)	44 (62.9)	43 (60.6)	
Male	156 (44.2)	102 (48.1)	26 (37.1)	28 (39.4)	
Years of education	5.26 (4.84)	6.42 (4.95)	6.73 (4.79)	5.32 (4.50)	0.166
Having a spouse					0.250
Yes	63 (17.8)	180 (84.9)	55 (78.6)	55 (77.5)	
No	290 (82.2)	32 (15.1)	15 (21.4)	16 (22.5)	
Economic condition					0.475
Good	131 (37.1)	128 (60.4)	46 (65.7)	48 (67.6)	
Poor	222 (62.9)	84 (39.6)	24 (34.3)	23 (32.4)	
Comorbidity					0.152
Yes	265 (75.1)	152 (71.7)	58 (82.9)	55 (77.5)	
No	88 (24.9)	60 (28.3)	12 (17.1)	16 (22.5)	
Body mass index					0.023
Underweight	20 (32.0)	8 (3.7)	6 (8.6)	6 (8.4)	
Normal	113 (5.7)	71 (33.5)	18 (25.7)	24 (33.8)	
Overweight	120 (34.0)	68 (32.1)	21 (30.0)	31 (43.7)	
Obese	100 (28.3)	65 (30.7)	25 (35.7)	10 (14.1)	
Leisure activities					
Active	6.71 (2.53)	6.99 (2.43)	6.69 (2.70)	5.89 (2.48)	0.006
Passive	13.61 (3.73)	14.27 (3.72)	13.37 (3.29)	11.87 (3.65)	<0.001
Physical function	3.70 (3.52)	4.11 (3.57)	3.56 (3.57)	2.61 (3.07)	0.007

**Table 2:** Bootstrap tests for the mediating effects of active and passive leisure activities (*n* = 353).

Effect	Mild Depression ^a^		Moderate/Severe Depression ^a^		
	Coefficient	95% CI	Coefficient	95% CI	Effect Size
Total indirect effect (c)	−0.025	−0.063, 0.015	−0.070 **	−0.119, −0.027	43%
Specific indirect effect (a × b)					
Active leisure activities	−0.014	−0.051, 0.018	−0.045 *	−0.083, −0.013	27%
Passive leisure activities	−0.010	−0.033, 0.001	−0.025	−0.062, 0.003	15%

Note: CI: confidence interval; ** *p* < 0.01, * *p* < 0.05, ^a^ Reference group: no depression.

## Data Availability

The datasets generated during the current study are not publicly available, but data are available from the applicants upon reasonable request and with permission of the researcher’s University.

## Supplementary Materials

The following supporting information can be downloaded at: https://www.mdpi.com/article/10.3390/ijerph19063543/s1. Figure S1: The mediating role of active and passive leisure activities in the association between depression and physical function (only adjusted for age and sex in the models, *n* = 353); Table S1: Bootstrap tests for the mediating effects of active and passive leisure activities (*n* = 353), adjusted for age and sex in the model; Figure S2: The mediating role of active and cognitive (passive) leisure activities in the association between depression and physical function (removed “watching television/listening to the radio/watching theatrical performances” from the category of passive leisure activities and renamed cognitive leisure activities, *n* = 353); Table S2: Bootstrap tests for the mediating effects of active and cognitive leisure activities (removed “watching television/listening to the radio/watching theatrical performances” from the category of passive leisure activities and reclassified them as cognitive leisure activities, *n* = 353), Figure S3: The mediating role of active and passive leisure activities in the association between depression and physical function (using depression as a continuous variable, *n* = 353); Table S3: Bootstrap tests for the mediating effects of active and passive leisure activities (using depression as a continuous variable, *n* = 353); Figure S4: The mediating role of active and passive leisure activities in the association between depression and physical function (using physical function as a dichotomous variable, *n* = 353); Table S4: Bootstrap tests for the mediating effects of active and passive leisure activities (using physical function as a dichotomous variable, *n* = 353).
