# Supplementary material for: Association between Depression Severity and Physical Function among Chinese Nursing Home Residents: The Mediating Role of Different Types of Leisure Activities
Source: Int J Environ Res Public Health. 2022 Mar 16;19(6):3543. doi: 10.3390/ijerph19063543 (PMC8955444; doi:10.3390/ijerph19063543)
Supplement: Supplementary file 1 [file ijerph-19-03543-s001.zip › ijerph-1614038-supplementary.pdf]

International Journal of
Environmental Research and Public Health

MDPI

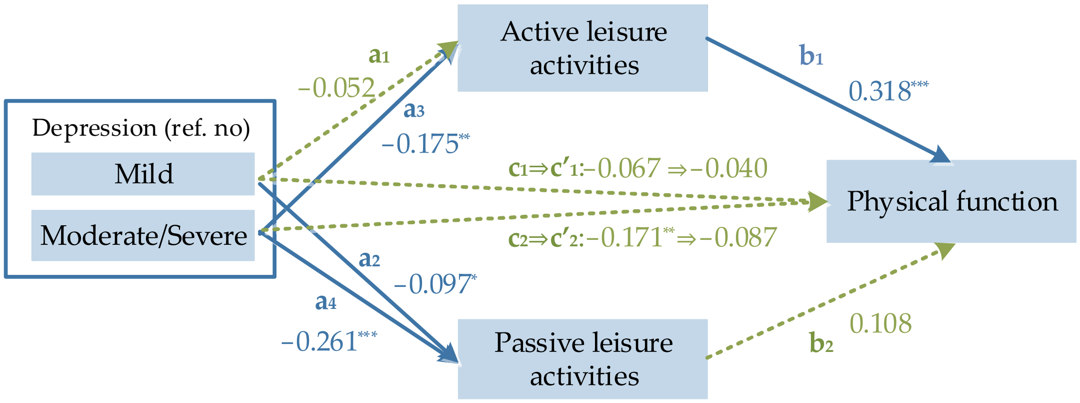

Figure S1. The mediating role of active and passive leisure activities in the association between depression and physical function (only adjusted for age and sex in the models, n = 353). Standardized coefficients are shown along the path arrows; solid lines represent statistical significance at $p < .05$, and dashed lines represent nonsignificant effects; Path a_1 represents the effect of mild depression on active leisure activities; Path a_2 represents the effect of mild depression on passive leisure activities; Path a_3 represents the effect of moderate/severe depression on active leisure activities; Path a_4 represents the effect of moderate/severe depression on passive leisure activities; Path b_1 represents the effect of active leisure activities on physical function; Path b_2 represents the effect of passive leisure activities on physical function; Path c_1 indicates the total effect of mild depression on physical function; Path c'_1 represents the direct effect of mild depression on physical function; Path c_2 indicates the total effect of moderate/severe depression on physical function; Path c'_1 represents the direct effect of moderate/severe depression on physical function; $^{*}p < .05$, $^{**}p < .01$, $^{***}p < .001$.

Table S1. Bootstrap tests for the mediating effects of active and passive leisure activities (n = 353), adjusted for age and sex in the model.

Effect	Mild [a]		Moderate/severe [a]		
	Coefficient	95% CI	Coefficient	95% CI	Effect size
Total indirect effect	−0.027	−0.069, 0.015	−0.084**	−0.135, −0.038	49%
Specific indirect effect					
Active leisure activities	−0.017	−0.056, 0.018	−0.056*	−0.095, −0.020	33%
Passive leisure activities	−0.011	−0.036, 0.001	−0.028	−0.069, 0.002	16%

Note: CI: confidence interval; $^{**}p < .01$, $^{*}p < .05$

[a] Reference group: no depression

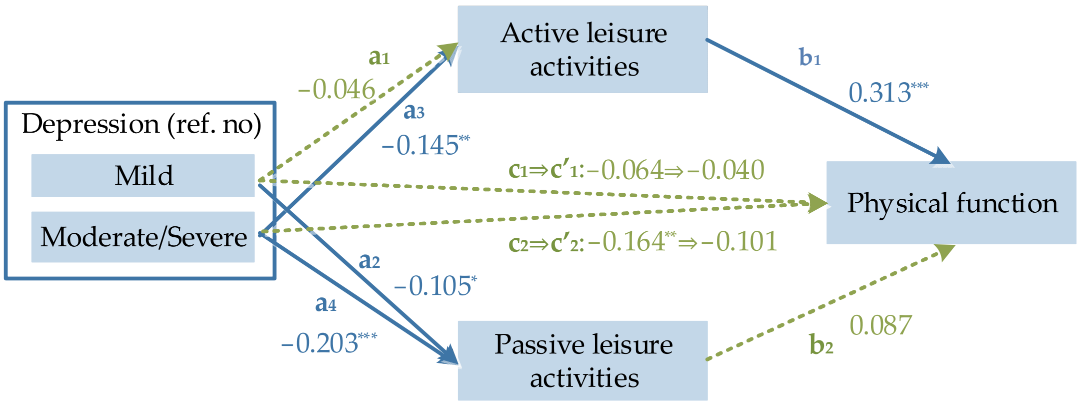

Figure S2. The mediating role of active leisure activities and cognitive (passive) leisure activities in the association between and physical function (removed "watching television/listening to the radio/watching theatrical performances" from the category of passive leisure activities and renamed cognitive leisure activities, $n = 353$). Standardized coefficients are shown along the path arrows; solid lines represent statistical significance at $p < .05$, and dashed lines represent nonsignificant effects; adjusted for age, sex, years of schooling, having a spouse, self−reported economic conditions, comorbidity conditions, and body mass index; Path a_1 represents the effect of mild depression on active leisure activities; Path a_2 represents the effect of mild depression on passive leisure activities; Path a_3 represents the effect of moderate/severe depression on active leisure activities; Path a_4 represents the effect of moderate/severe depression on passive leisure activities; Path b_1 represents the effect of active leisure activities on physical function; Path b_2 represents the effect of passive leisure activities on physical function; Path c_1 indicates the total effect of mild depression on physical function; Path c'_1 represents the direct effect of mild depression on physical function; Path c_2 indicates the total effect of moderate/severe depression on physical function; Path c'_1 represents the direct effect of moderate/severe depression on physical function; $^{*}p < .05$, $^{**}p < .01$, $^{***}p < .001$.

Table S2. Bootstrap tests for the mediating effects of active leisure activities and cognitive leisure activities (removed "watching television/listening to the radio/watching theatrical performances" from the category of passive leisure activities and reclassified them as cognitive leisure activities, $n = 353$)

Effect	Mild [a]		Moderate/severe [a]		
	Coefficient	95% CI	Coefficient	95% CI	Effect size
Total indirect effect	−0.023	−0.064, 0.016	−0.063**	−0.111, −0.021	38%
Specific indirect effect					
Active leisure activities	−0.014	−0.051, 0.018	−0.045*	−0.084, −0.013	27%
Cognitive leisure activities	−0.009	−0.032, 0.002	−0.018	0.051, 0.005	11%

Note: CI: confidence interval; $^{**}p < .01$, $^{*}p < .05$

[a] Reference group: no depression

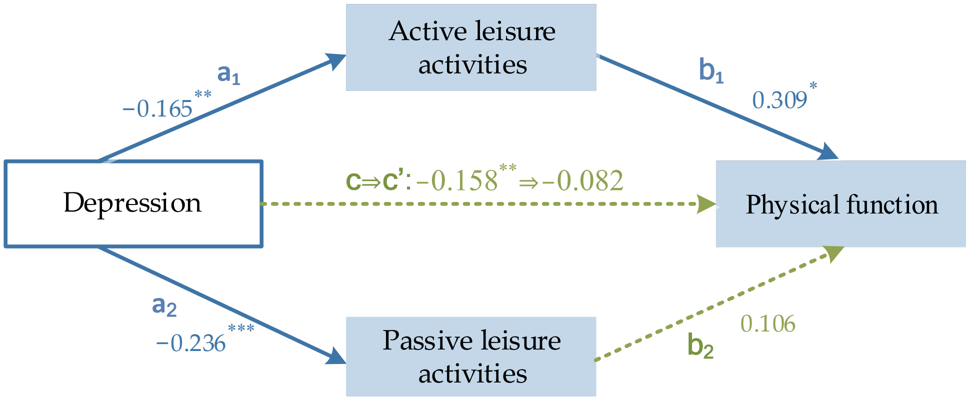

Figure S3. The mediating role of active and passive leisure activities in the association between and physical function (using depression as a continuous variable, $n = 353$). Standardized coefficients are shown along the path arrows; solid lines represent statistical significance at $p < .05$, and dashed lines represent nonsignificant effects; adjusted for age, sex, years of schooling, having a spouse, self–reported economic conditions, comorbidity conditions, and body mass index; Path a_1 represents the effect of depression on active leisure activities; Path a_2 represents the effect of depression on passive leisure activities; Path b_1 represents the effect of active leisure activities on physical function; Path b_2 represents the effect of passive leisure activities on physical function; Path c indicates the total effect of depression on physical function; Path c' represents the direct effect of depression on physical function; $^{*}p < .05$, $^{**}p < .01$, $^{***}p < .001$.

Table S3. Bootstrap tests for the mediating effects of active and passive leisure activities (using depression as a continuous variable, $n = 353$)

Effect	Bootstrapped distribution		
	Coefficient	95% CI	Effect size
Total indirect effect	−0.076**	−0.122, −0.033	48%
Specific indirect effect			
Active leisure activities	−0.051*	−0.060, −0.002	32%
Passive leisure activities	−0.025	−0.091, 0.019	16%

Note: CI: confidence interval; $^{**}p < .01$, $^{*}p < .05$

[a] Reference group: no depression

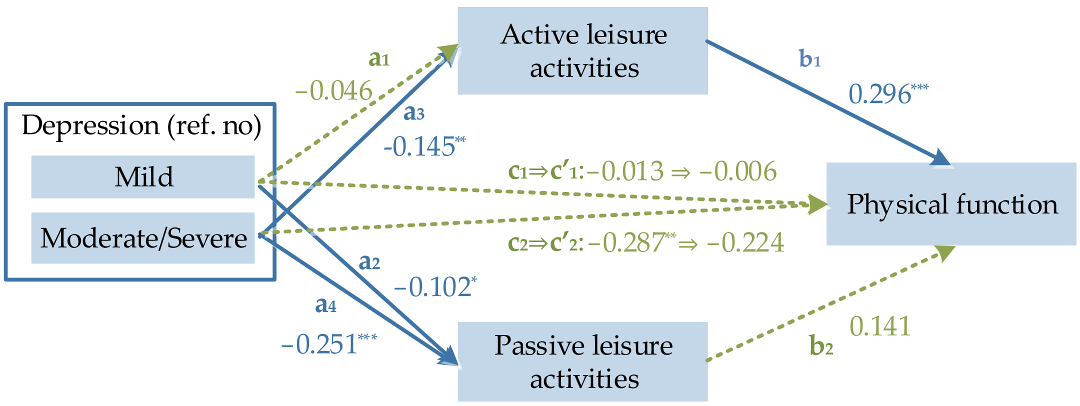

Figure S4. The mediating role of active and passive leisure activities in the association between depression and physical function (using physical function as the dichotomous outcome, $n = 353$). Standardized coefficients are shown along the path arrows; solid lines represent statistical significance at $p < .05$, and dashed lines represent nonsignificant effects; adjusted for age, sex, years of schooling, having a spouse, self−reported economic conditions, comorbidity conditions, and body mass index; Path a_1 represents the effect of mild depression on active leisure activities; Path a_2 represents the effect of mild depression on passive leisure activities; Path a_3 represents the effect of moderate/severe depression on active leisure activities; Path a_4 represents the effect of moderate/severe depression on passive leisure activities; Path b_1 represents the effect of active leisure activities on physical function; Path b_2 represents the effect of passive leisure activities on physical function; Path c_1 indicates the total effect of mild depression on physical function; Path c'_1 represents the direct effect of mild depression on physical function; Path c_2 indicates the total effect of moderate/severe depression on physical function; Path c'_1 represents the direct effect of moderate/severe depression on physical function; $^{*}p < .05$, $^{**}p < .01$, $^{***}p < .001$.

Table S4. Bootstrap tests for the mediating effects of active and passive leisure activities (using physical function as a dichotomous variable, $n = 353$)

Effect	Mild depression[a]		Moderate/severe depression[a]		
	Coefficient	95% CI	Coefficient	95% CI	Effect size
Total indirect effect	0.028	−0.003, 0.088	0.078**	0.034, 0.161	27%
Specific indirect effect					
Active leisure activities	0.014	−0.010, 0.062	0.043*	0.015, 0.103	15%
Passive leisure activities	0.014	−0.001, 0.050	0.035	−0.002, 0.099	12%

Note: CI: confidence interval; $^{**}p < .01$, $^{*}p < .05$

[a] Reference group: no depression
